# RNA-Seq Reveals Dynamic Changes of Gene Expression in Key Stages of Intestine Regeneration in the Sea Cucumber *Apostichopus japonicas*

**DOI:** 10.1371/journal.pone.0069441

**Published:** 2013-08-06

**Authors:** Lina Sun, Hongsheng Yang, Muyan Chen, Deyou Ma, Chenggang Lin

**Affiliations:** 1 Institute of Oceanology, Chinese Academy of Sciences, Qingdao, PR China; 2 University of Chinese Academy of Sciences, Beijing, PR China; 3 Ocean University of China, Qingdao, PR China; Cankiri Karatekin University, Turkey

## Abstract

**Background:**

Sea cucumbers (Holothuroidea; Echinodermata) have the capacity to regenerate lost tissues and organs. Although the histological and cytological aspects of intestine regeneration have been extensively studied, little is known of the genetic mechanisms involved. There has, however, been a renewed effort to develop a database of Expressed Sequence Tags (ESTs) in *Apostichopus japonicus*, an economically-important species that occurs in China. This is important for studies on genetic breeding, molecular markers and special physiological phenomena. We have also constructed a library of ESTs obtained from the regenerative body wall and intestine of *A. japonicus*. The database has increased to ∼30000 ESTs.

**Results:**

We used RNA-Seq to determine gene expression profiles associated with intestinal regeneration in *A. japonicus* at 3, 7, 14 and 21 days post evisceration (dpe). This was compared to profiles obtained from a normally-functioning intestine. Approximately 5 million (M) reads were sequenced in every library. Over 2400 up-regulated genes (>10%) and over 1000 down-regulated genes (∼5%) were observed at 3 and 7dpe (log_2_Ratio≥1, FDR≤0.001). Specific “Go terms” revealed that the DEGs (Differentially Expressed Genes) performed an important function at every regeneration stage. Besides some expected pathways (for example, Ribosome and Spliceosome pathway term), the “Notch signaling pathway,” the “ECM-receptor interaction” and the “Cytokine-cytokine receptor interaction” were significantly enriched. We also investigated the expression profiles of developmental genes, ECM-associated genes and Cytoskeletal genes. Twenty of the most important differentially expressed genes (DEGs) were verified by Real-time PCR, which resulted in a trend concordance of almost 100% between the two techniques.

**Conclusion:**

Our studies demonstrated dynamic changes in global gene expression during intestine regeneration and presented a series of candidate genes and enriched pathways that contribute to intestine regeneration in sea cucumbers. This provides a foundation for future studies on the genetics/molecular mechanisms associated with intestine regeneration.

## Introduction

Regeneration, which can be described as the regrowth, or repair, of cells, tissues and organs, is a widespread phenomenon found among certain metazoans [1], [2]. Besides its obvious advantages in terms of survival, regeneration also enhances the adaptive capacities of a species within its natural environment [3]. A considerable amount of research has focused on the fundamental biology of regeneration, including cell dedifferentiation, transdifferentiation, proliferation and migration. There is, however, a need for further studies into key aspects of regeneration, such as the target genes associated with the regenerative processes, and the molecular mechanisms involved [2], [4].

Echinodermata possess spectacular regenerative capacity [1], [3]. The sea cucumbers (Holothuroidea) are capable of regenerating most tissues and organs, including the intestine, respiratory tree, gonads and the body wall. They are thus considered as excellent models for organ regeneration studies [1], [5], [6], [7]. Regeneration has been the topic of considerable research, with an emphasis on visceral (intestine) regeneration, including the histological changes (for example, tissue layer changes in the intestine wall and changes in the radial nerve cord) and cellular events (cell origin, migration and proliferation) associated with such regeneration [8], [9], [10], [11], [12], [13], [14]. Nevertheless, due to the lack of information on the genome of sea cucumbers, only relatively few genes – such as Ependymin, Wnt9, Bmp1 and Serum amyloid A *etc.* – have been screened, recognized and analyzed, using traditional methods [11], [15], [16]. A greater effort on the study of the molecular/genetic mechanisms involved in intestine regeneration in the Holothuroidea is therefore needed.

Pablo et al. constructed an EST library (∼5000 ESTs) and used a microarray technique to analyze gene expression profiles during intestine regeneration in the cucumber *Holothuria glaberrima* [7], [10]. However, approximately 5000 ESTs generated by Sanger sequencing were not nearly sufficient to cover the transcriptome of sea cucumber. Moreover, because of limitations due to lower throughput, high background noise and lower sensitivity of microarray, it's hard to obtain high-quality global gene expression profiles. The development of an ultrahigh-throughput sequencing RNA-Seq technique, however, provided a powerful alternative technology for gene expression profiling [17], [18]. The RNA-Seq technique has the potential to overcome microarray limitations and provide an expression profile with a greater and reproducible dynamic range. RNA-Seq has been successfully applied to many research projects, including the transcriptional landscape of the yeast genome, research on sulfur-deprived *Chlamydomonas* cell survival and mapping, as well as the quantification of mammalian transcriptomes [19], [20], [21].

In a previous study, our team constructed a transcriptome of the regenerative body wall (after 4 days of regeneration) and intestine (7dpe) in *A. japonicus* by 454 life sequencing which, to a large extent, increased the EST information required for a molecular mechanism study on regeneration [22]. This research was, however, of a preliminary nature, in contrast with the present study on intestine regeneration in *A. japonicus,* which includes five key stages: wound healing (0–3dpe), blastema formation (3–7dpe), lumen formation (7–14dpe), intestine differentiation (14–21dpe) and growth (21dpe-) [23]. The following processes took place during different stages: Stage I: activation of damage repair capacity in response to injury, so as to accumulate energy in preparation for regeneration [24]; Stage II: formation of the blastema, which encompasses cell migration, dedifferentiation and transdifferentiation; Stage III: luminal epithelium growth by cell division and migration, which results in lumen formation of new intestine; Stage IV: gradual development of the intestine to form a complete structure in which the digestive and absorptive functions are restored. Stage V: enlargement of the intestine to reach its original normal size. This process can thus be described as a continuous dynamic change in the gene expression that underlies the molecular mechanisms involved in regeneration.

In the present study, we used RNA-Seq to determine the global dynamic changes in the gene expression profile, occurring during the intestine regeneration in *A. japonicus*. For robustness, the reference library, which integrated nine transcriptomes, (in all ∼30,000 isotigs), included all tissues of sea cucumbers at every developmental stage (embryo, larva, white juvenile and black juvenile) and under different physiological conditions, i.e. active, regeneration and aestivation conditions [22], [25]. Based on this premise, we compared the expression profiles of the intestine in a Normal situation (with intact intestine) with those recorded at 3, 7, 14, 21 days post evisceration (dpe). The global dynamic changes in gene expression during each of these stages were analyzed. The sampling time covered all stages of intestine regeneration. As expected, a total of up to 5119 DEGs associated with intestine regeneration were identified. Furthermore, gene ontology (GO) and pathways enrichment analysis were conducted for investigating the main function of DEGs and providing an overview of the gene regulation process during intestine regeneration. For example, “organic substance transport” was only observed at 3dpe, which indicated that during the early stage of the process sufficient nutritional and energy resources were assembled to enable the commencement of regeneration. Besides the expected pathways, the enriched “Notch signaling pathway”, “ECM-receptor interaction” and “Cytokine-cytokine receptor interaction” were substantially activated during intestine regeneration. It should be noted that little overlap was observed between the screened top DEGs when comparing our results with those of Pablo et al. To sum up, our work may provide a more representative basis for the future study of molecular mechanisms associated with intestine regeneration.

## Materials and Methods

### Ethics Statement

Not applicable. Our research did not involve human participants or samples.

### Animals

Adult sea cucumbers, *A. japonicus* (70–100 g), were collected from the coast of Qingdao, Shandong Province and cultured in a laboratory for 1 week in sea water at 15–17°C, prior to setting up the experiment. Evisceration was induced by injecting about 2 ml 0.35 M KCl into the coelom [6], [10], [16]. The non-eviscerated sea cucumbers (control) were fed once a day. All sea cucumbers were anesthetized in 6% MgCl_2_ for about 1 h before being sacrificed. 15 individuals per stage, at 3, 7, 10, 14 and 21 days post evisceration (dpe) were used for our experiments. 15 non-eviscerated sea cucumbers served as the control. The dissected normal and regenerative intestines were frozen and stored in liquid nitrogen.

### RNA preparation and cDNA synthesis

Total RNA from regenerative (from animals at 3 dpe, 7 dpe, 14 dpe, 21 dpe) and normal intestines (control) was extracted and DNase-treated using RNeasy Mini Kit and RNase-Free DNase Set (Qiagen, Germany), following the manufacturer' s instructions. The quality and concentration of RNA were measured by NanoDrop 1000 (Thermo). Total RNA from 15 individuals per stage were pooled. The starting amount of RNA was 1ug per pool (including 15 individuals). After that, the mRNA in total RNA was enriched by using the oligo(dT) magnetic beads. After the addition of fragmentation buffer, the mRNA was interrupted and formed short fragments (∼200 bp). The first strand cDNA was then synthesized by the random hexamer-primer, using the mRNA fragments as templates. Buffer, dNTPs, RNase H and DNA polymerase I were added to synthesize the second strand. The double strand cDNA was purified using the QiaQuick PCR extraction kit, and washed with EB buffer so as to facilitate end repair and the addition of a single nucleotide A (adenine). Finally, sequencing adaptors were ligated to the fragments. The required fragments were purified by means of agarose gel electrophoresis and enriched by PCR amplification. The library products were then ready for sequencing analysis via Illumina HiSeq™ 2000 (BGI, Shenzhen).

### Sequence annotation, assessment and gene expression levels

The original image data was transferred into sequence data by base calling (using CASAVA version 1.5+, defined as ‘raw reads’) and saved as fastq files that include the detailed reads sequences and the reads quality information. Specifically, if the sequencing error rate is denoted as E, Illunima HiSeq™ 2000, and the base quality value is denoted as sQ, the relationship is as follows: sQ  = −10lgE. To obtain clean reads, it is necessary to remove the dirty raw reads prior to data analysis. This process encompasses the following procedures: (1) remove reads with adaptors; (2) remove reads in which unknown bases are greater than 10%; (3) remove low-quality reads (the percentage of the low quality bases of quality value ≤5 is more than 50% in a read).

A comparison of “clean reads” by SOAPaligner/soap2 was carried out using the reference databases from large-scale transcriptome profiling [22], [25]. Assessment of sequencing was conducted in five steps: sequence reads quality assessment, statistics of alignment analysis, sequencing saturation analysis, distribution of reads on reference genes, and gene coverage analysis. Mismatches of no more than two bases were allowed in the alignment. The number of clean tags was calculated and normalized, using the RPKM method (Reads Per kb Million reads) [21].

### Screening of differentially expressed genes (DEGs)

According to the Audic et al.'s algorithm, a strict algorithm to identify differentially expressed genes between two samples was developed [26]. The P value was used to detect the difference in gene expression in two different samples [27]. If the P value is less than1E-238 (P<1E–238 ), the P value is given the value “0”. The FDR (False Discovery Rate) was estimated, to determine the threshold of P-value. The “FDR≤0.001 and the absolute value of log_2_Ratio≥1” was used as the threshold to judge the significance of gene expression difference. All DEGs were screened by program “stastistic” written using Language C. Previous studies have demonstrated that 48 genes differentially expressed responding to the injection [28]. However, the intestine regeneration may involve the regulation of immune genes. Hence, These genes were not subtracted from the regeneration profile.

### Gene ontology and Pathway enrichment analysis

Gene Ontology (GO) is an international standardized gene functional classification system which offers a dynamic-updated controlled vocabulary and a strictly defined concept to comprehensively describe properties of genes and their products in any organism. GO has three ontologies: molecular function, cellular component and biological process. The basic unit of GO is GO-term. Pathway-based analysis helps to further understand genes biological functions. KEGG is the major public pathway-related database. The first stage is to map all DEGs to GO terms in the database (http://www.geneontology.org/) using program Blast2GO and KEGG (http://www.genome.jp/kegg/) using KEGG Automatic Annotation Server (KAAS) and calculated gene numbers, after which the hypergeometric test is used to find significantly-enriched GO terms and pathways in DEGs. GO terms conforming to p-value through Bonferroni Correction≤0.05 were defined as significantly enriched GO terms and pathways [29].

### Real-time PCR validation

To validate RNA-seq results, some significant DEGs were chosen to carry out Real-time PCR. According to the sequence information in the transcriptome database, primers were designed for optimal performance using the primer3 (Table S1). The RNA that was used to synthesize cDNA was the same as that used to RNA-seq. The first strand cDNA was synthesized in 25 μl reaction system as follows. Firstly, 4 μl RNA and 1μl oligodT18 was degenerated at 70°C for 5 min. Then, 1 μl M-MLV reverse transcriptase (Promega), 5 μl M-MLV buffer (25 mmol/L KCl, 10 mmol/L Tris-HCl, 0.6 mmol/L MgCl2, and 2 nmol/L DTT, pH 8.3), 5 μl dNTP, 1 μl ribonuclease inhibitor and 8 μl RNase-free water were added at 42°C for 1 h. The synthesized cDNAs were diluted with RNase-free water and stored at −80°C for subsequent quantitative real-time PCR.

The mRNA expression levels were determined using the SYBR Green® real-time PCR assay with an Eppendorf Mastercycler®ep realplex (Eppendorf, Hamburg, Germany). The amplification volume was 25 μL, containing 12.5 μL of SYBR GreenMasterMix (Takara), 0.5 μL (each) of forward and reverse primer (10 μM), 1 μL of diluted cDNA, and 10.5 μL of RNase-free water. Thermal cycling was as follows: (1) 95°C for 5 s; (2) 40 cycles at 95°C for 10 s, 60°C for 20 s and 72°C for 30 s. The melting curve analysis of the amplification products was done to demonstrate the specificity of the PCR products. NADH dehydrogenase (NADHF, NADHR, Table S1) was used as a housekeeping gene for internal standardization [7], [16], [22], [30]. The 2−△△CT method was used to analyze the expression level. Five biological pools (N = 5), with each pool (3 dpe, 7 dpe, 14 dpe, 21 dpe and Normal) being mixed with three different sea cucumber individuals (n = 3 animals) were analyzed, because of the small quantity of regenerative intestine. All data were given as mean ± S.E. (N = 5) and the level of statistical significance was set at P<0.05. Analysis was carried out using SPSS16 software.

## Results

### Reads sequencing, quantification and assembly

A total of 4 868 208, 4 727 453, 5 030 570, 4 715 682 and 4 877 984 reads were sequenced using RNA-Seq technique in Normal, 3dpe, 7dpe, 14dpe and 21dpe libraries, respectively, which have been submitted to NCBI (accession NO. GSE44995; Table 1). After trimming the dirty raw reads (reads with adaptors, more than 10% unknown bases and over 50% bases of quality value ≤5), 4 848 595, 4 708 854, 5 011 237, 4 696 545 and 4 855 872 clean reads were obtained in Normal, 3dpe, 7dpe, 14dpe and 21dpe libraries, respectively. (Table 1). The map reference transcriptome from 454 life sequencing contains nine library- embryos (4 h, 23 h); larvae (30 h, 6d,8d, 10d); white juveniles (16d, 22d); black juveniles (32d, 37d); female gonads; male gonads; intestine, respiratory trees and coelomic fluids from active adults; intestine, respiratory trees and coelomic fluid from aestivating adults; and regenerating intestine and body wall. Therefore there are abundant expressed sequence tags (29 667 ESTs) in the reference transcriptome to cover the whole transcriptome [22], [25]. In the present study, 2 332 657, 2 084 836, 2 276 114, 2 230 612 and 2 395 628 reads in five libraries – which accounted for 48.11%, 44.27%, 45.42%, 47.49% and 49.33% of total clean reads, respectively – were mapped to 22691 isotigs (unigenes) from the reference transcriptome data. Only perfect match reads and ≤2 mismatch reads were counted. Further analysis revealed that 53.6% (1 250 409/2 332 657), 62.3% (1 298 097/2 084 836), 62.9% (1 431 558/2 276 114), 61.7% (1 376 454/2 230 612) and 55.5% (1 328 904/2 395 628) unique reads in five libraries matched to only one gene in sea cucumber transcriptome.

**Table 1:** Summary alignment statistics in Normal, 3dpe, 7dpe, 14dpe and 21dpe libraries.

	Normal		3dpe		7dpe		14dpe		21dpe	
Map to Gene	readsnumber	percentage	reads number	percentage	reads number	percentage	reads number	percentage	reads number	percentage
**Total Reads**	4868208	100.00%	4727453	100.00%	5030570	100.00%	4715682	100.00%	4877984	100.00%
**Total clean Reads**	4848595	99.6%*	4708854	99.61%	5011237	99.62%	4696545	99.59%	4855872	99.55%
**Total BasePairs**	2.38 E+08		2.3 E+08		2.46E+08		2.3E+08		2.38E+08	
**Total Mapped Reads**	2332657	48.11%#	2084836	44.27%	2276114	45.42%	2230612	47.49%	2395628	49.33%
**perfect match**	1687851	34.81%#	1476471	31.36%	1603772	32.00%	1588510	33.82%	1735483	35.74%
**< = 2bp mismatch**	644806	13.30%#	608365	12.92%	672342	13.42%	642102	13.67%	660145	13.59%
**unique match**	1250409	25.79%#	1298097	27.57%	1431558	28.57%	1376454	29.31%	1328904	27.37%
**multi-position match**	1082248	22.32%#	786739	16.71%	844556	16.85%	854158	18.19%	1066724	21.97%

### Sequencing Saturation Analysis

Saturation of the library is related to the depth of sequencing. When barely any new genes are detected, sequencing reaches saturation. With the number of reads increasing, the number of detected genes increased. The more reads were sequenced, the better results were achieved. Figure S1 indicates that, the percentage of detected genes was less than 2% when the number of reads increased from 4 to 5M. ∼5 M reads sequenced in each library provide sufficient coverage depth to achieve good results. Sequencing saturation analysis also presented the transcriptome size in the five libraries: R3 = R7>R14>R21>Normal.

### Differentially expressed genes (DEGs)

**Figure 1:**
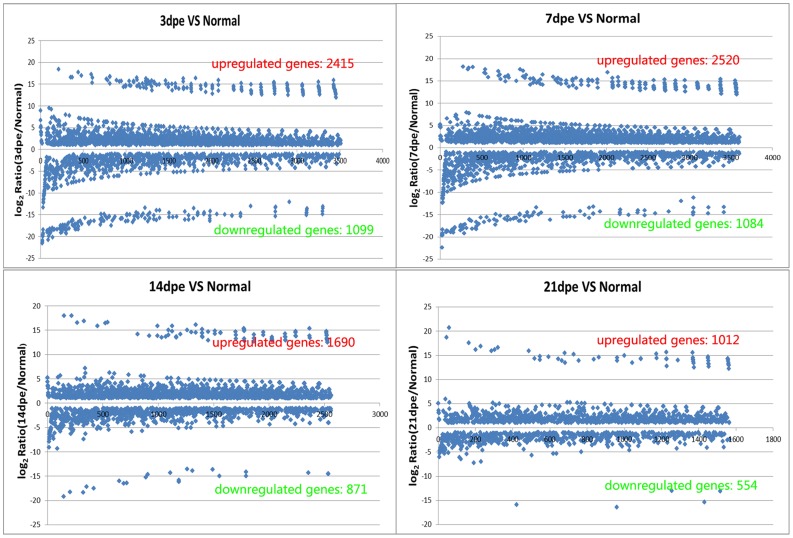
The change level of global differential expression genes during intestine regeneration in sea cucumber *A. japonicus*. The principle "FDR≤0.001 and the absolute value of log2Ratio≥1” was used as a threshold to screen DEGs. The figure showed that not only a large number of genes were differentially expressed, but the change fold of differentially expression was at a high level, especially at the early stage of intestine regeneration.

The RNA-seq results showed sequential large-scale genes expression profiles at 3dpe, 7dpe, 14dpe and 21dpe during intestine regeneration in *A. japonicus*. The transcripts detected with at least two-fold differences (log_2_Ratio≥1) are classified as DEGs (FDR≤0.001) (Figure 1). Scatterplot was applied, to graphically describe the expression profile of global differential genes. A large number of genes were differentially expressed at a high level between normal and regenerative libraries, especially at the early stage of intestine regeneration (Figure 1). For example, when compared to Normal, 2415 up-regulated genes (10.64%, 2415/22691) and 1099 (4.84%, 1099/22691), the down-regulated genes were observed at 3dpe; and 2520 (11.11%, 2520/22691) up-regulated genes and 1084 (4.78%, 1084/22691) down-regulated genes were screened at 7dpe. The number and expression level of DEGs gradually decreased in the 21dpe library, and only 1012 (4.46%, 1012/22691) up-regulated genes and 554 (2.44%, 554/22691) down-regulated genes were observed. In addition, we also developed a strategy to focus on key regeneration genes by comparing gene expression between two libraries (3dpe and 7dpe) during the early stages of regeneration. Only 280 up-regulated genes and 256 down-regulated were observed at 3dpe, in comparison with 7dpe (Table S2).

**Figure 2:**
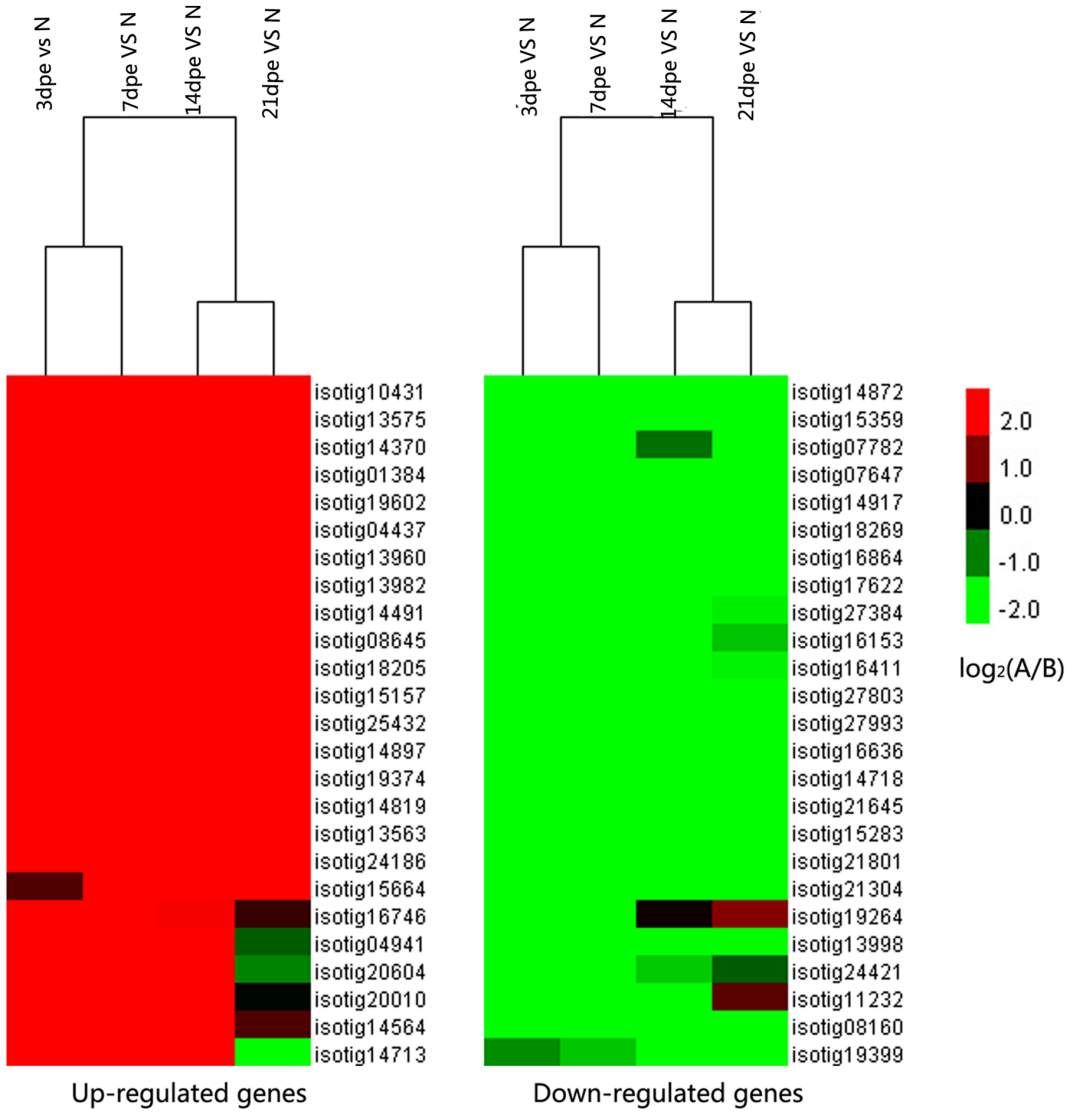
The expression pattern of top significantly differentially expressed genes at 3dpe, 7dpe, 14dpe and 21dpe. Expression differences were shown in different colors. Red mean up regulation and green mean down regulation.

There were occasional No Reads observations in some libraries, resulting in the high change fold of DEGs between two libraries. In such cases, though, it was difficult to assess the real difference of DEGs. In addition, some mapped genes with no reference information in the NCBI database could not be annotated. These unknown genes were hard to analyze and in such situations we here showed the representative top 10 DEGs, based on the criteria (Tables 2,3,4,5 and 6). When the number of reads mapping to one gene is 0 in one library, the counterpart must be more than 100 in the other library. As shown on the top DEGs list, most DEGs – such as low density lipoprotein-related protein 2-like, orthodenticle, cyclin B3 Hu/elav isoform 7, Rp2 Lipase, proprotein convertase subtilisin/kexin type 9 – were significantly differentially expressed throughout the intestine regeneration (Figure 2, Tables 2,3,4 and 5). Some special DEGs–kelch-like ECH-associated protein 1, HORMA domain containing 1-like and Human Fc gamma BP et al – were screened at 3dpe Vs 7dpe (Table 6). Unknown DGEs with high change fold were also notable, since they might correspond to novel sea cucumbers-specific sequences associated with their striking regenerative capacities. Unknown DEGs with high change fold can be found in Table S3, S4, S5, S6 for further comprehensive and specific research.

**Table 2:** Top 10 differentially expressed genes at 3 dpe VS Normal.

Top	Gene ID	Blast X	Log_2_(3dpe/N)	P value	FDR
1↑	isotig19602	low density lipoprotein-related protein 2-like	18.4758	1.09E-114	1.08E-112
2↑	isotig14370	GL12416-like isoform 2	16.5964	1.74E-60	1.02E-58
3↑	isotig13575	speedy A	15.2978	3.45E-31	1.18E-29
4↑	isotig16746	regeneration associated protein	8.9914	0	0
5↑	isotig14713	orthodenticle	7.1358	1.25E-41	5.44E-40
6↑	isotig14897	solute carrier family 6 member 9 transcript-like	6.8885	5.79E-35	2.16E-33
7↑	isotig20604	cyclin B3	6.7021	0	0
8↑	isotig14819	TFP250	6.6430	1.98E-114	1.94E-112
9↑	isotig04941	Hu/elav isoform 7	6.5754	6.05E-109	5.61E-107
10↑	isotig20010	cleavage stage histone H1	6.3723	2.83E-24	7.86E-23
1↓	isotig27803	Rp2 Lipase	−21.5166	0	0
2↓	isotig24421	cellular retinol-binding protein type 1b	−21.4169	0	0
3↓	isotig19264	fatty acid binding protein 2, intestinal	−21.0933	0	0
4↓	isotig27993	proprotein convertase subtilisin/kexin type 9	−20.7676	2.40E-262	5.75E-260
5↓	isotig16864	alpha-amylase	−19.7367	0	0
6↓	isotig17622	FG-GAP repeat family protein	−19.7265	0	0
7↓	isotig13998	LOC495367 protein	−19.6356	0	0
8↓	isotig14917	triacylglycerol lipase, pancreatic	−19.4072	0	0
9↓	isotig18269	lysozyme	−19.0389	5.26E-194	9.19E-192
10↓	isotig16411	cytosolic beta-glucosidase-like	−18.4674	6.44E-161	9.03E-159

↑: up-regulated ↓:down-regulated FDR: False Discovery Rate “0” means P value <1E-238.

**Table 3:** Top 10 differentially expressed genes at 7 dpe VS Normal.

Top	Gene ID	Blast X	Log_2_(7dpe/N)	P value	FDR
1↑	isotig19602	low density lipoprotein-related protein 2-like	16.7534	3.84E-36	1.50E-34
2↑	isotig01384	H3.3 histone	15.7117	3.83E-33	1.38E-31
3↑	isotig24186	insulin-like growth factor-binding protein 7	7.6874	5.39E-63	3.29E-61
4↑	isotig16746	regeneration associated protein	7.2505	6.74E-137	7.28E-135
5↑	isotig13563	C-type lectin superfamily 4	6.9748	4.02E-38	1.66E-36
6↑	isotig04941	Hu/elav isoform 7	6.9238	5.33E-144	5.94E-142
7↑	isotig14713	orthodenticle	6.5053	2.35E-27	7.22E-26
8↑	isotig20010	cleavage stage histone H1	6.4772	8.11E-27	2.44E-25
9↑	isotig20604	cyclin B3	6.4630	2.58E-281	6.34E-279
10↑	isotig14564	Nek protein	6.2311	1.58E-22	4.13E-21
1↓	isotig15359	proprotein convertase subtilisin/kexin type 9	−22.4921	0	0
2↓	isotig16864	alpha-amylase	−19.7367	0	0
3↓	isotig17622	FG-GAP repeat family protein	−19.7265	0	0
4↓	isotig14917	triacylglycerol lipase, pancreatic	−19.4072	0	0
5↓	isotig14872	preamylase 1	−19.2917	0	0
6↓	isotig21304	cholinesterase 1	−19.1335	8.90E-169	1.19E-166
7↓	isotig18269	lysozyme	−19.0389	6.98E-208	1.13E-205
8↓	isotig16411	cytosolic beta-glucosidase-like	−18.4674	2.01E-172	2.74E-170
9↓	isotig16153	Phospholipase	−18.3292	1.76E-162	2.27E-160
10↓	isotig11232	cellular retinol-binding protein type 1b	−18.2508	6.56E-89	5.04E-87

↑: up-regulated ↓:down-regulated FDR: False Discovery Rate “0” means P value <1E-238.

**Table 4:** Top 10 differentially expressed genes at 14 dpe VS Normal.

Top	Gene ID	Blast X	Log_2_(14dpe/N)	P value	FDR
1↑	isotig01384	H3.3 histone	16.5670	8.33E-59	6.31E-57
2↑	isotig10431	S-crystallin SL11	15.8891	3.54E-30	1.57E-28
3↑	isotig15664	elongation of very long chain fatty acids	6.2278	1.65E-43	9.71E-42
4↑	isotig20010	cleavage stage histone H1	6.1282	9.76E-21	3.13E-19
5↑	isotig24186	insulin-like growth factor-binding protein 7	5.8156	1.29E-16	3.40E-15
6↑	isotig20604	cyclin B3	5.6735	1.51E-154	2.66E-152
7↑	isotig14713	orthodenticle	5.6163	1.99E-14	4.56E-13
8↑	isotig04941	Hu/elav isoform 7	5.3771	1.20E-45	7.29E-44
9↑	isotig08645	intrinsic factor-B12 receptor precursor	5.3732	3.65E-178	7.97E-176
10↑	isotig13563	C-type lectin superfamily 4, member G	5.1834	1.27E-10	2.25E-09
1↓	isotig11232	cellular retinol-binding protein type 1b	−18.2508	1.68E-86	1.64E-84
2↓	isotig08160	MGC68755 protein	−17.1262	1.09E-41	6.21E-40
3↓	isotig07647	proprotein convertase subtilisin/kexin type 9	−9.3060	0	0
4↓	isotig21801	LOC495367 protein	−7.8665	0	0
5↓	isotig27384	polyhydroxybutyrate depolymerase-like	−7.3480	1.46E-46	8.97E-45
6↓	isotig16864	alpha-amylase	−7.3456	0	0
7↓	isotig27803	Rp2 Lipase	−6.9528	0	0
8↓	isotig14872	preamylase 1	−6.4091	0	0
9↓	isotig17622	FG-GAP repeat family protein	−6.1212	0	0
10↓	isotig14718	triacylglycerol lipase, pancreatic	−5.9002	0	0

↑: up-regulated ↓:down-regulated FDR: False Discovery Rate “0” means P value <1E-238.

**Table 5:** Top 10 differentially expressed genes at 21 dpe VS Normal.

Top	Gene ID	Blast X	Log_2_(21dpe/N)	P value	FDR
1↑	isotig01384	H3.3 histone	16.1434	1.26E-43	1.02E-41
2↑	isotig08645	intrinsic factor-B12 receptor precursor	5.1410	2.67E-147	8.61E-145
3↑	isotig04437	mCG4790	5.0954	1.14E-27	6.34E-26
4↑	isotig14491	OSJNBa0008M17.5	4.9996	2.99E-09	6.20E-08
5↑	isotig13982	cubilin-like	4.9250	3.79E-55	3.81E-53
6↑	isotig13960	tektin 3	4.9122	4.40E-24	2.17E-22
7↑	isotig19374	low density lipoprotein-related protein 2-like	4.8191	3.78E-08	6.86E-07
8↑	isotig15157	monocarboxylate transporter MCT2	4.6126	4.72E-07	7.39E-06
9↑	isotig18205	Leucine zipper transcription factor-like 1	4.4971	1.65E-06	2.37E-05
10↑	isotig25432	neurogenic locus notch homolog protein 2	4.4668	9.01E-12	2.38E-10
1↓	isotig08160	MGC68755 protein	−7.0765	7.53E-39	5.30E-37
2↓	isotig07782	Natterin-3	−6.5252	7.10E-77	1.04E-74
3↓	isotig15283	proprotein convertase subtilisin/kexin type 9	−6.1150	0	0
4↓	isotig16636	peroxisomal bifunctional enzyme	−5.5208	1.85E-60	2.10E-58
5↓	isotig19399	tenascin XB-like	−5.4098	5.67E-12	1.52E-10
6↓	isotig27803	Rp2 Lipase	−5.3172	0	0
7↓	isotig14872	preamylase 1	−5.0173	8.00E-304	6.35E-301
8↓	isotig14718	triacylglycerol lipase, pancreatic	−4.7723	0	0
9↓	isotig21801	LOC495367 protein	−4.7714	0	0
10↓	isotig21645	Sea star regeneration-associated protease	−4.6698	0	0

↑: up-regulated ↓:down-regulated FDR: False Discovery Rate “0” means P value <1E-238.

**Table 6:** Top 10 differentially expressed genes at 3dpe vs 7dpe.

Top	Gene ID	Blast X	Log2(3dpe/N)	P value	FDR
1↑	isotig01386	H3.3 histone	3.6648	3.44E-06	9.89E-05
2↑	isotig21606	LOC495367 protein	3.6006	2.57E-08	1.09E-06
3↑	isotig16741	calcium activated chloride channel	3.5887	1.49E-52	7.01E-50
4↑	isotig20235	kelch-like ECH-associated protein 1	2.7850	1.93E-05	4.64E-3
5↑	isotig19861	CMP -N-acetylneuraminic acid	2.4917	1.70E-16	1.63E-14
6↑	isotig11169	ribosomal protein L23a	2.4340	2.08E-08	8.97E-07
7↑	isotig16483	solute carrier family 5	2.4138	6.34E-87	5.49E-84
8↑	isotig14600	HORMA domain containing 1-like	2.2395	3.91E-23	5.76E-21
9↑	isotig26466	Human Fc gamma BP	2.1856	1.79E-05	4.33E-3
10↑	isotig19556	attractin-like 1	2.1412	2.96E-06	8.64E-05
1↓	isotig11231	cellular retinol-binding protein type 1b	−17.4466	7.03E-51	3.18E-48
2↓	isotig15821	annexin A7	−6.5848	2.96E-194	5.83E-19
3↓	isotig08881	lactase-phlorizin hydrolase	−5.9207	0	0
4↓	isotig15664	elongation of very long chain fatty acids	−4.5593	2.20E-24	3.43E-22
5↓	isotig03622	Glucosamine-6-phosphate isomerase	4.2511	1.56E-05	3.83E-3
6↓	isotig24215	tetraspanin 11	4.1807	2.86E-05	6.56 E-3
7↓	isotig10431	S-crystallin SL11	4.1807	2.86E-05	6.56 E-3
8↓	isotig08953	26S proteasome non-ATPase regulatory subunit	3.7168	9.39E-07	3.04E-05
9↓	isotig28826	structural maintenance of chromosomes protein	3.6137	3.01E-06	8.75E-05
10↓	isotig22672	multidrug resistance protein (MRP5)	3.5593	1.77E-08	7.73E-07

↑: up-regulated ↓:down-regulated FDR: False Discovery Rate.

The genes unique to a certain stage are very important. 386 genes that were unique to 3dpe were found on Table S8, 117 of which could be annotated, such as f-box only protein 47-like, Notch homolog Scalloped wings-like and heat shock protein 70 et al (Table S8). Similarly, 366 (125 genes could be annotated such as vesicle-associated protein and cathepsin L-associated protein et al ), 248 (75 genes could be annotated such as DnaJ homolog, Transmembrane protein 30A and major yolk protein 1 et al )and 229 genes (71 genes could be annotated such as novel EGF domain containing protein et al )found on Table S8 were unique to 7, 14 and 21 dpe respectively (Table S8). Though so many genes were unique to a time point, these genes were low expressed. only one or a few unique reads could be mapped to them.

### Validation of Results by real-time PCR

**Figure 3:**
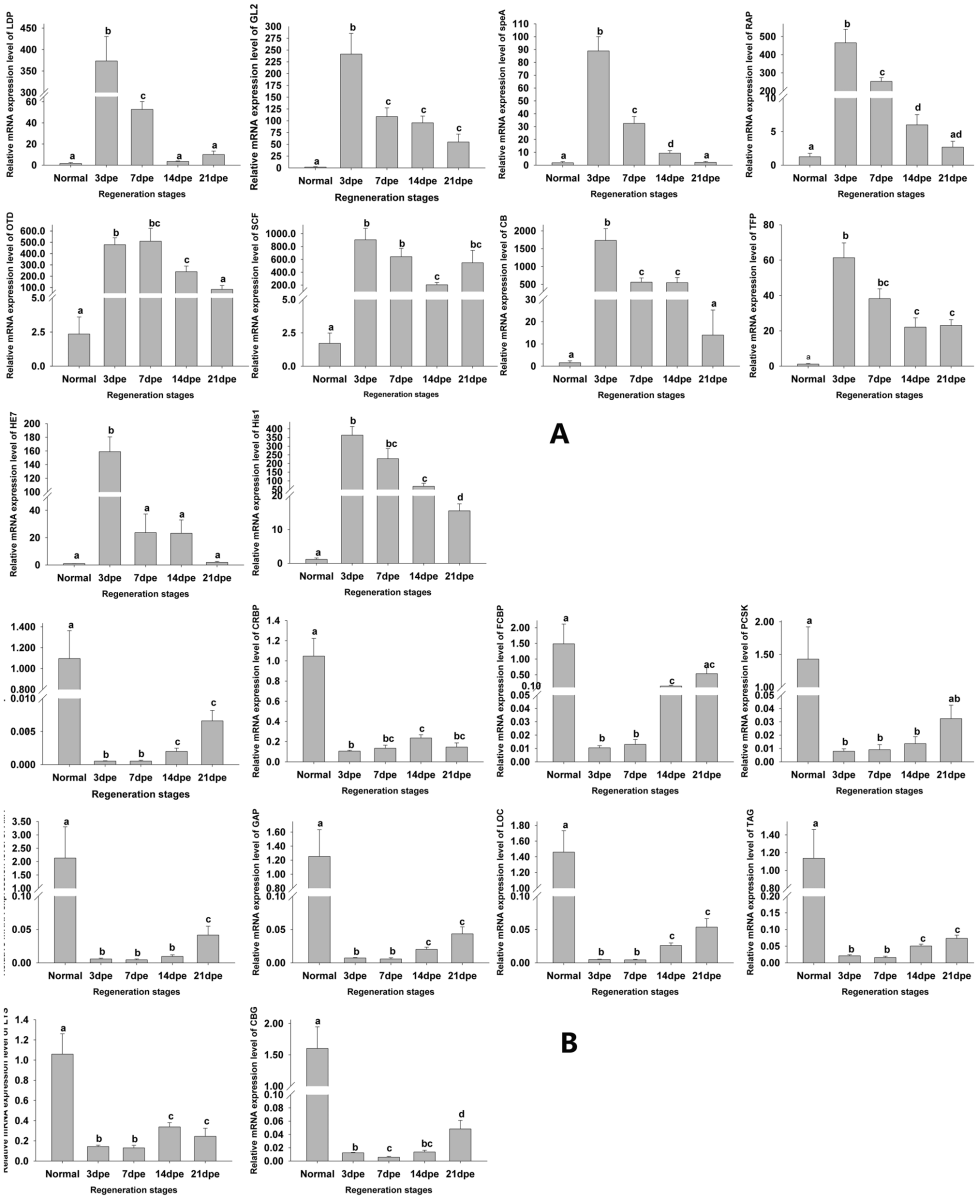
Real-time PCR analysis for top 10 up-regulated and 10 down-regulated genes. (A). LDP: low density lipoprotein-related protein 2-like; GL 2: GL12416-like isoform 2; speA: speedy A; RAP: regeneration associated protein; OTD: orthodenticle SCF: solute carrier family 6 member 9 transcript-like; CB: cyclin B3 TFP: TFP250 HE7: Hu/elav isoform 7; his1: cleavage stage histone H1. (B). Rp2: Rp2 Lipase; CRBP: cellular retinol-binding protein type 1b; FCBP: fatty acid binding protein 2, intestinal; PCSK: proprotein convertase subtilisin/kexin type 9; AMY: alpha-amylase; GAP: FG-GAP repeat family protein; LOC: LOC495367 protein; TAG: triacylglycerol lipase, pancreatic; LYS: lysozyme; CBG: cytosolic beta-glucosidase-like. Different lowercase letters indicate significant differences (P<0.05). Values indicate the mean ± S.E. (N = 5).

To validate the expression profiles, top 10 up-regulated genes (abbreviation: LDP, GL2, speA, RAP, OTD, SCF, CB, TFP, HE7 and His1) and top 10 down-regulated genes (abbreviation: Rp2, CRBP, FCBP, PCSK, AMY, GAP, LOC, TAG, LYS, CBG), selected from Table 2, were applied to real-time PCR at four stages of regeneration (normal, 3dpe, 7dpe and 21dpe) (Table 7; Figure 3). NADH dehydrogenase was taken as a reference gene to normalize gene expression data, based on a previous investigation [7], [16], [22], [30]. All primers were tested to be effective on PCR amplication. One peak in the melting curve was detected in all real-time PCR, which indicated that the all PCR product was specifically amplified (data not shown). Real-time PCR results indicated that the expression level of these selected genes reached its highest at 3dpe. The overall trend of Real-time PCR-based expression patterns among these selected genes was similar to those obtained by RNA-Seq based detection. However, the changes fold of most data measured by Real-time PCR was smaller than those by RNA-seq (Table 7).

**Table 7:** Genes selected for Real-time PCR validation.

		3dpe (change fold)		7dpe (change fold)		14dpe (change fold)		21dpe (change fold)	
Gene ID	Gene	RNA-seq	RT-PCR	RNA-seq	RT-PCR	RNA-seq	RT-PCR	RNA-seq	RT-PCR
isotig19602	LDP	3.65E+05	373.44±57.02	1.10E+05	52.78±7.45	1.68E+04	3.554±0.44	28.229	10.065±3.26
isotig14370	GL2	9.91E+04	466.24±44.18	2.29E+04	108.83±18.54	5.95E+03	95.604±14.39	474.464	54.918±16.84
isotig13575	speA	4.03E+04	56.37±11.19	2.84E+04	47.47±5.47	1.31E+04	9.29±1.99	378.331	2.19± 0.71
isotig16746	RAP	508.96	790.49±74.41	152.27	178.09±20.70	7.57	6.00±1.52	1.568	2.68±0.87
isotig14713	OTD	140.63	879.25±61.11	90.84	609.50±114.05	49.05	240.33±49.51	0.002	83.24±34.77
isotig14897	SCF	118.48	830.92±175.86	46.29	541.73±130.10	13.63	904.29±36.41	8.468	796.85±194.50
isotig20604	CB	104.12	4732.38±334.08	88.22	4061.21±117.27	51.04	1.00E+03±144.50	0.342	13.954±11.30
isotig14819	TFP	99.94	61.37±8.40	57.43	38.19±5.59	18.40	22.08±5.30	21.171	20.58±3.30
isotig04941	HE7	95.37	156.50±21.60	121.41	23.65±13.66	41.56	23.22±9.72	0.470	1.91±0.75
isotig20010	His1	82.84	239.41±49.89	89.09	252.52±59.60	69.95	68.79±17.42	0.941	13.04±2.06
isotig27803	Rp2	3.33E-07	5.28E-04±4.86E-05	5.97E-04	5.29E-04±1.24E-04	8.07E-03	1.99E-03±4.80E-04	2.51E-02	6.60E-03±1.58E-03
isotig24421	CRBP	3.57E-07	1.06E-01±7.80E-03	3.21E-06	1.34E-01±0.03	3.21E-06	2.35E-01±0.03	4.69E-01	1.45E-01±0.04
isotig19264	FCBP	4.47E-07	1.05E-02±1.64E-03	2.50E-02	1.30E-02±3.59E-03	1.13E+00	1.38E-01±0.03	2.99E+00	5.34E-01±0.19
isotig27993	PCSK	5.60E-07	7.91E-03±1.83E-03	1.70E-07	9.09E-03±3.78E-03	1.58E-03	1.36E-02±5.23 E-03	1.44E-02	3.24E-02±0.01
isotig16864	AMY	1.14E-06	5.70E-03±1.23E-03	1.14E-06	4.59E-03±1.23E-03	6.15E-03	9.65E-03±2.35 E-03	4.78E-02	4.15E-02±0.01
isotig17622	GAP	1.15E-06	7.30E-03±5.37E-04	1.15E-06	5.78E-03±1.70E-03	1.44E-02	2.02E-02±3.29 E-03	4.55E-02	4.34E-02±0.01
isotig13998	LOC	1.23E-06	5.00E-03±5.24E-04	9.93E-04	4.38E-03±8.68E-04	4.28E-03	2.59E-02±3.87 E-03	1.25E-01	5.35E-02±0.01
isotig14917	TAG	1.44E-06	2.11E-02±3.16E-03	1.44E-06	1.60E-02±3.67E-03	1.67E-02	5.04E-02±5.64 E-03	3.66E-02	7.33E-02±9.81E-03
isotig18269	LYS	1.86E-06	1.43E-01±0.01	1.86E-06	1.30E-01±0.03	3.49E-02	3.38E-01±0.04	1.07E-01	2.45E-01±0.08
isotig16411	CBG	2.76E-06	1.24E-02±6.01 E-04	2.76E-06	5.77E-03±1.24 E-03	7.89E-02	1.35E-02±2.65 E-03	1.36E-01	4.84E-02±0.01

### Gene ontology analysis of DEGs

**Figure 4:**
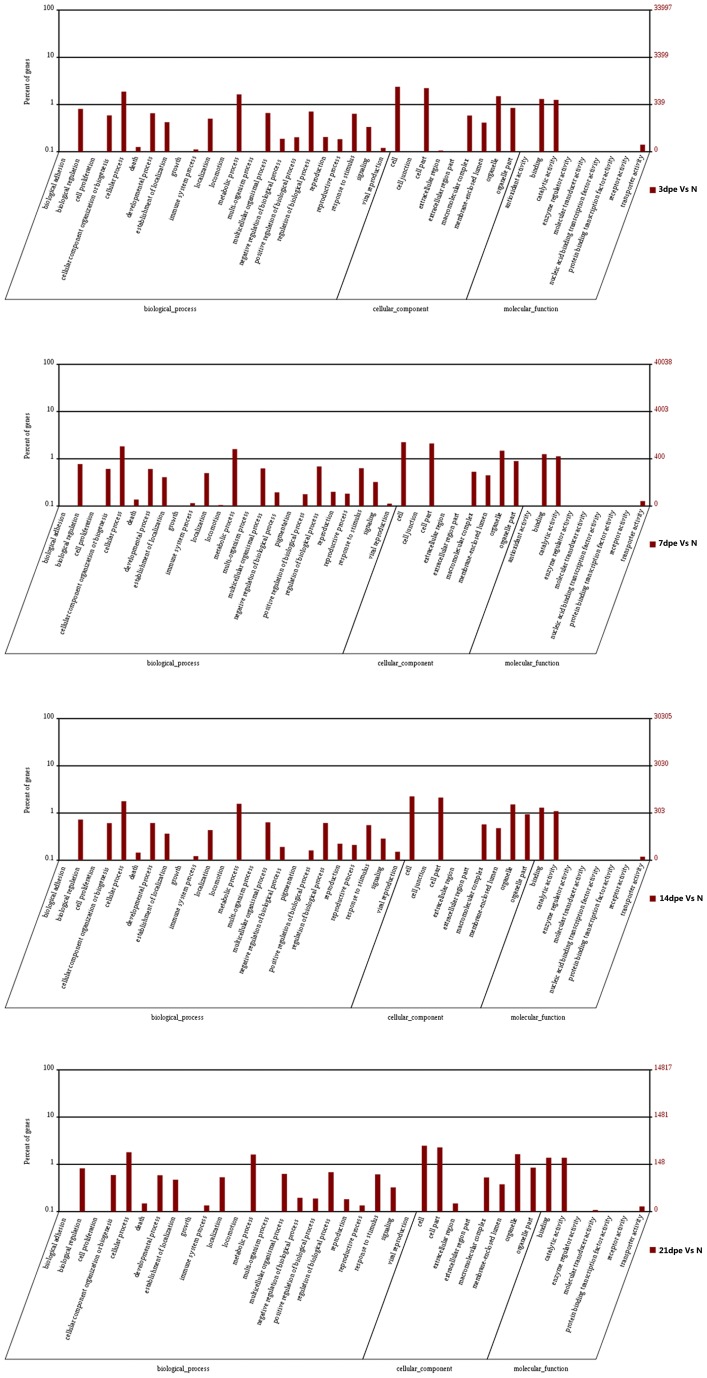
Distribution of gene ontology (GO) terms of differentially expressed genes during intestine regeneration. The percentage of GO-terms in the categories “Molecular function”,“Biological Process” and “Cellular component” was shown.

DEGs, that were involved in certain biological functions during intestine regeneration, were evaluated by GO (gene ontology) analysis. GO terms were assigned to 1111(31.6%, 1111/3514), 1230 (34.1%, 1230/3604), 905 (35.3%, 905/2561) and 498 (31.8%, 498/1566) DEGs for 3, 7, 14 and 21dpe, respectively (Figure 4). The DEGs associated with cellular and metabolic processes in the category ‘biological process’; the DEGs associated with cell and cell part in the category ‘cellular component’ and the DEGs associated with binding and catalytic activity in the category ‘molecular function’ were notably abundant throughout the intestine regeneration. The GO distribution of the DEGs was highly similar at different regeneration stages. Moreover, we screened the significantly enriched GO terms classified in biological function category. As indicated in Table 8 , some GO terms – ‘pancreas development’, ‘gene expression’, ‘translation’ and ‘reproduction’ – were significantly enriched at 3, 7 and 14dpe. At 3dpe, only six GO terms (pancreas development, gene expression, translation, reproduction, organic subtance transport and reproductive process) were significantly enriched, which were mainly associated with development (4) and transport (1). GO term ‘organic subtance transport’ was only enriched at 3dpe. 15 GO terms (ribonucleoprotein complex biogenesis, cellular component biogenesis at cellular level, ribosome biogenesis and primary metabolic process et al)were significantly enriched at 7dpe, which were associated with development (5) and metabolism (10) GO term ‘organ development’ was only enriched at 7dpe. In particular, as much as 28 GO terms were significantly enriched at 14dpe, which were associated with development (4), metabolism (14), cell cycle (6), signal transduction (2) and transport(2). 14 GO term (cell cycle, proteasomal protein catabolic process and signal transduction in response to DNA damage et al) were only enriched at 14dpe (Table 8, corrected p-value≤0.05). No significantly enriched GO terms in DEGs were detected at 21 dpe. Besides, two unique GO terms-” serine family amino acid metabolic process” and “polyol metabolic process” were significantly enriched at 3dpe Vs 7dpe (Table 8).

**Table 8:** Significantly enriched GO terms in DEGs.

			R3 VS Normal		R7 VS Normal		R14 VS Normal	
Gene Ontology term	reference (N/M)		Cluster (n/m)	P-value	Cluster (n/m)	P-value	Cluster (n/m)	P-value
pancreas development^4^	65/5270 (1.2%)		47/842 (5.6%)	3.79E-21	50/952 (5.3%)	3.63E-22	47/698 (6.7%)	6.84E-25
gene expression	754/5270 (14.3%)		171/842 (20.3%)	0.00013	207/952 (21.7%)	3.75E-09	170/698 (24.4%)	1.71E-11
translation	191/5270 (3.6%)		57/842 (6.8%)	0.00081	71/952 (7.5%)	1.79E-07	69/698 (9.9%)	2.34E-13
Reproduction^4^	264/5270 (5.0%)		71/842 (8.4%)	0.00275	78/952 (8.2%)	0.00238	65/698 (9.3%)	0.00021
organic substance transport^5^	94/5270 (1.8%)		33/842 (3.9%)	0.00428				
reproductive process^4^	263/5270 (5.0%)		70/842 (8.3%)	0.00487	77/952 (8.1%)	0.00408	65/698 (9.3%)	0.00018
RC biogenesis^2^	92/5270 (1.7%)				41/952 (4.3%)	3.50E-06	38/698 (5.4%)	1.58E-08
CCB^2^	92/5270 (1.7%)				41/952 (4.3%)	3.50E-06	38/698 (5.4%)	1.58E-08
ribosome biogenesis^2^	64/5270 (1.2%)				29/952 (3.0%)	0.00048	28/698 (4.0%)	1.65E-06
primary metabolic process^2^	2426/5270 (46.0%)				502/952 (52.7%)	0.00342	381/698 (54.6%)	0.00076
CMBP^2^	555/5270 (10.5%)				140/952 (14.7%)	0.00613	120/698 (17.2%)	5.32E-06
MBP^2^	561/5270 (10.6%)				141/952 (14.8%)	0.00698	120/698 (17.2%)	1.05E-05
MMP^2^	1902/5270 (36.1%)				403/952 (42.3%)	0.00791	328/698 (47.0%)	1.48E-07
organ development^4^	560/5270 (10.6%)				138/952 (14.5%)	0.02682		
CMMP^2^	1671/5270 (31.7%)				355/952 (37.3%)	0.0366	283/698 (40.5%)	7.82E-05
metabolic process^2^	3160/5270 (60.0%)				625/952 (65.7%)	0.0475	468/698 (67.0%)	0.02166
mitotic cell cycle^1^	190/5270 (3.6%)						53/698 (7.6%)	4.06E-05
protein metabolic process^2^	1020/5270 (19.4%)						189/698 (27.1%)	5.89E-05
CPMP^2^	922/5270 (17.5%)						173/698 (24.8%)	0.0001
cellular biosynthetic process^2^	742/5270 (14.1%)						145/698 (20.8%)	0.00012
cell cycle^1^	369/5270 (7.0%)						84/698 (12.0%)	0.00014
biosynthetic process^2^	770/5270 (14.6%)						148/698 (21.2%)	0.00027
cell cycle phase^1^	233/5270 (4.4%)						59/698 (8.5%)	0.00029
cell cycle process^1^	303/5270 (5.7%)						69/698 (9.9%)	0.00208
PPCP^2^	51/5270 (1.0%)						20/698 (2.9%)	0.00305
STRDD^3^	47/5270 (0.9%)						19/698 (2.7%)	0.00315
Interphase^1^	74/5270 (1.4%)						25/698 (3.6%)	0.0045
organ development^4^	560/5270 (10.6%)						109/698 (15.6%)	0.00808
DSTP53^3^	23/5270 (0.4%)						12/698 (1.7%)	0.00929
RULA^1^	36/5270 (0.7%)						15/698 (2.1%)	0.02286
PUDPC^ 2^	49/5270 (0.9%)						18/698 (2.6%)	0.02763
**R3 Vs R7**								
Gene Ontology term		reference frequency (N/M) Cluster frequency(n/m) Corrected P-value						
serine family amino acid metabolic process^2^		19/5270(0.4%) 6/134(4.5%) 0.00205						
polyol metabolic process^2^		11/5270(0.2%) 6/134(3.0%) 0.04719						

N is the number of all genes with GO annotation; n is the number of DEGs in N; M is the number of all genes that are annotated to the certain GO terms; m is the number of DEGs in M. There were no significantly enriched GO terms in DEGs at 21 dpe.

1 Cell cycle ^2^ Metabolism ^3^ Signal transduction ^4^development ^5^ transport

GO terms with corrected p-value≤0.05 were significantly enriched in DEGs.

RC biogenesis: ribonucleoprotein complex biogenesis CCB: cellular component biogenesis at cellular level.

CMBP: cellular macromolecule biosynthetic process MBP: macromolecule biosynthetic process.

MMP: macromolecule metabolic process CMMP: cellular macromolecule metabolic process.

CPMP: cellular protein metabolic process PPCP: proteasomal protein catabolic process.

STRDD: signal transduction in response to DNA damage.

DSTP53: DNA damage response, signal transduction by p53 class mediator.

RULA: regulation of ubiquitinprotein ligase activity involved in mitotic cell cycle.

PUDPC: proteasomal ubiquitin -dependent protein catabolic process.

### Pathway enrichment analysis of DEGs

Intestine regeneration associated biological pathways were evaluated by enrichment analysis of DEGs. Significantly enriched metabolic pathways and signal transduction pathways were identified, and listed in Table 9 (Q value <0.05). Seven significantly enriched pathways for the up-regulated DEGs were screened. “Cytokine-cytokine receptor interaction” and “Notch signaling pathway”, “Ribosome”, “Spliceosome”, “RNA transport”, “DNA replication” and “Ribosome biogenesis in eukaryotes”, were related to the biogenesis of protein, DNA and RNA. There were eight significantly enriched pathways for the down-regulated DEGs, which were involved in digestion and absorption of vitamin fats and carbohydrates, the Renin-angiotensin system, Pancreatic secretion, and metabolism of Glycerophospholipid, alpha-Linolenic and Starch and sucrose. In addition, “Retinol metabolism”, “Protein digestion and absorption”, “PPAR signaling pathway”, “Glutathione metabolism” and “ECM-receptor interaction”, including some up-regulated and down-regulated DEGs, were also enriched. For example, DEGs for adipocyte differentiation were up-regulated (12) and DEGs for lipid metabolism were down-regulated (15) in the PPAR signaling pathway. Only one unique pathway” Glycine, serine and threonine metabolism” were found at 3dpe Vs 7dpe. The DEGs in enriched pathways were listed in Table S7.

**Table 9:** Significantly enriched pathway in DEGs.

		R3 VS N	R7 VS N	R14 VS N	R21 VS N
Pathway ID	Pathway term	Q value	Q value	Q value	Q value
Pathways for up-regulated DEGs					
ko03010	Ribosome	3.35E-42	1.76E-40	4.90E-53	1.67E-05
ko04060	Cytokine-cytokine receptor interaction	3.56E-02			
ko04330	Notch signaling pathway	4.14E-02			
ko03040	Spliceosome	4.73E-02	1.97E-05	5.04E-04	1.76E-02
ko03013	RNA transport		1.73E-03		
ko03030	DNA replication		7.67E-03	3.15E-02	
ko03008	Ribosome biogenesis in eukaryotes			2.58E-03	
Pathways for down-regulated DEGs					
ko04977	Vitamin digestion and absorption	6.66E-10	7.74E-08	1.09E-06	1.03E-11
ko04614	Renin-angiotensin system	7.30E-07	7.85E-04	9.54E-03	1.97E-04
ko00564	Glycerophospholipid metabolism	2.86E-05	8.94E-04	3.15E-04	3.33E-07
ko00592	alpha-Linolenic acid metabolism	5.94E-04	6.26E-05	5.04E-04	4.53E-05
ko04975	Fat digestion and absorption	3.78E-03	1.73E-03	1.27E-02	2.02E-03
ko04973	Carbohydrate digestion and absorption	2.63E-02			5.30E-03
ko04972	Pancreatic secretion	2.63E-02	3.40E-02	1.80E-02	5.30E-03
ko00500	Starch and sucrose metabolism	2.63E-02			
Pathways for some up-regulated DEGs and some down-regulated DEGs					
ko00830	Retinol metabolism	3.34E-03	1.73E-03	4.03E-02	
ko04974	Protein digestion and absorption	7.92E-03	2.18E-02	3.15E-02	1.29E-03
ko03320	PPAR signaling pathway		4.59E-02		3.50E-03
ko00480	Glutathione metabolism			2.59E-02	1.97E-04
ko04512	ECM-receptor interaction			2.64E-02	1.97E-04
**R3 Vs R7** Q value					
ko00260	Glycine, serine and threonine metabolism	3.43E-03			

Pathways with Q≤0.05 were deemed significantly enriched in DEGs.

### Individual key DEGs

Key genes associated with the regenerative process were classified into three groups: developmental genes, extracellular matrix (ECM) associated genes, and cytoskeletal genes.

#### a. Developmental genes

Regeneration and development share similar mechanisms, so developmental genes are excellent candidates for future study on molecular mechanisms of regeneration [4]. The expression profiles of well-known developmental genes are therefore summarized in Table 10. Most genes (Wnt, Hox, BMP and syndecan) were up-regulated; while krueppel like6 were down-regulated during regeneration. In addition, genes from the same family showed different expression patterns. For example, *Hox1* and *Hox*3 were up-regulated at early stage 3 ,7 14 dpe and reached a peak at 3dpe. No changes were observed for *Hox9/10* at 3dpe, but its expression remained at a high level from 7dpe to 21dpe. In the case of *Hox11/13*, the up-regulating trends occurred from 14dpe.

**Table 10:** Expression profile of developmental genes during intestine regeneration.

Gene ID	Gene name	Normal(RPKM)	3dpe(RPKM)	7dpe(RPKM)	14dpe(RPKM)	21dpe(RPKM)
isotig19205	Wnt4	4.68	14.43↑	45.81↑	20.42↑	12.34↑
isotig18112	Wnt6	5.97	9.04↑	21.62↑	16.28↑	6.42
isotig14310	Wnt8	–	1.93	–	1.36	–
isotig24437	Hox1	8.77	33.80↑	26.82↑	18.59↑	12.38
isotig16174	Hox3	–	19.62↑	5.34↑	4.32↑	–
isotig23550	Hox9/10	2.75	3.97	21.60↑	22.47↑	21.98↑
isotig18972	Hox 11/13	–	–	0.80	1.67	3.46↑
isotig17483	BMP	21.38	22.88	48.41↑	48.91↑	39.49↑
isotig14688	BMP1	6.96	31.45↑	30.86↑	24.31↑	19.64↑
isotig03217	syndecan	170.41	271.40↑	214.40	172.68	219.38
isotig28523	krueppel-like6	31.99	19.81↓	21.95↓	20.76↓	47.30↑

RPKM: normalized gene expression level ↑: up-regulated ↓:down-regulated.

#### b. ECM-associated genes

Quinones *et* *al.* have found that, in the sea cucumber *H. glaberrima,* the ECM content undergoes significant changes during intestine regeneration and that those changes are closely related to MMPs activity [13]. In this study six family genes (collagen, tenascin, laminin, MMPs, spondin and fibropellin) associated with ECM were screened. All of these genes, except MNP14, up-regulated at one or several stages of intestine regeneration (Table 11).

**Table 11:** Expression profile of ECM associated genes during intestine regeneration.

Gene ID	Gene name	Normal(RPKM)	3dpe(RPKM)	7dpe(RPKM)	14dpe(RPKM)	21dpe(RPKM)
isotig22120	alpha-2 collagen	13.41	63.41↑	86.25↑	110.75↑	95.21↑
isotig06513	alpha-5 collagen	43.46	148.89↑	236.37↑	265.37↑	299.74↑
isotig00683	tenascin R	–	2.26↑	4.09↑	5.68↑	0.73
isotig16822	tenascin XB-like	–	4.36↑	0.66	2.06↑	0.71
isotig15853	laminin alpha	12.97	107.46↑	96.88↑	58.33↑	25.63↑
isotig20868	laminin gamma	63.63	262.07↑	318.13↑	174.40↑	149.67↑
isotig07199	MMP1	0.35	1.36	3.08↑	1.60↑	0.33
isotig15379	MMP12	–	0.58	1.06↑	1.10↑	–
isotig19398	MMP14	6.67	0.92↓	2.50↓	6.06	5.38
isotig14227	MMP16	31.79	181.87↑	96.13↑	31.55	39.58
isotig13187	MMP19	4.18	12.37↑	51.13↑	47.75↑	57.60↑
isotig23432	MMP20	–	5.21↑	–	–	–
isotig22633	spondin	11.48	28.26↑	69.07↑	34.76↑	52.81↑
isotig09128	fibropellin Ia	0.57	1.09	2.47↑	1.54↑	1.07↑
isotig17742	fibropellin Ib	9.06	26.18↑	35.97↑	39.65↑	38.75↑
isotig19172	papilin	140.30	–	0.82↓	14.45↓	24.64↓

RPKM: normalized gene expression level ↑: up-regulated ↓:down-regulated.

#### c. Cytoskeletal genes

Changes in cytoskeletal protein synthesis during regeneration have been demonstrated in many regeneration tissues [31], [32], [33]. All cytoskeletal genes showed significant changes at 3 or 7dpe (Table 12). Some (alpha- tubulin, beta-tubulin and actin) were up-regulated; while others (gamma tubulin, myosin and gelsolin) were down-regulated.

**Table 12:** Expression profile of Cytoskeletal genes during intestine regeneration.

Gene ID	Gene name	Normal(RPKM)	3dpe(RPKM)	7dpe(RPKM)	14dpe(RPKM)	21dpe(RPKM)
isotig14228	alpha-tubulin	23.05	60.93↑	71.10↑	81.96↑	88.12↑
isotig22271	Beta-tubulin	24.57	57.98↑	115.89↑	152.89↑	97.10↑
isotig23490	gamma-tubulin	23.12	9.17↓	23.76	18.53	16.64
isotig02880	actin	103.15	82.45	186.76↑	209.20↑	255.78↑
isotig07384	actin 2	73.99	144.74↑	230.59↑	244.80↑	321.89↑
isotig08506	myosin III	4.06	0.36↓	0.32↓	1.01↓	0.35↓
isotig15711	myosin V	33.06	22.05↓	23.88↓	20.21↓	38.28
isotig16090	myosin VI	63.12	12.94↓	20.53↓	27.45↓	21.48↓
isotig15703	gelsolin	1500.62	373.58↓	489.86↓	758.17↓	1014.86↓

RPKM: normalized gene expression level ↑: up-regulated ↓:down-regulated.

## Discussion

In this study, an RNA-Seq technique was used to construct the large-scale dynamic expression profile during intestine regeneration in *A. japonicus*. Real-time PCR was used to verify gene expression profiles. We found that the results of the verifications were comparable to the gene expression profiles. They had similar trends of up- and down-regulation. However, the change scales of these gene expressions, especially for down-regulated genes, were larger for RNA-Seq, compared with those for Real-time PCR. The RNA-Seq was probably more sensitive in terms of determining gene expression levels, particularly for low-abundance transcripts [17]. The individual variation may be another reason for the difference in change fold. For RNA-Seq assay, 15 individuals were pooled to run; while for Real-time PCR, 5 samples were used separately.

Thousands of genes (more than 15%, ∼3500ESTs) showed significantly differential expression during intestine regeneration. This was to be expected, as intestine regeneration is a very complicated process that includes epithelial cell migration and proliferation, contraction of injured area, changes in cellular function, and communication and interaction of multiple cell types. [13], [34], [35]. This result is not completely consistent with a report by Pablo *et* *al* in which ∼2000 ESTs – a third of the total – showed a different expression in *H. glaberrima.* [7]. Besides which, there was almost no overlap in top DEGs between these two studies. The difference scales of top DEGs were much larger in our results than Pablo *et* *al*. The number of ESTs matched to known genes in the reference libraries was also different: only ∼1500 ESTs (∼20%) in the *H. glaberrima* library and ∼13000 ESTs (47%) in the *A. japonicus* library. The reasons for these differences might be due to three factors. First, most *H. glaberrima* ESTs were only obtained from regenerative intestine, so the original data was biased in favour of the genes associated with regeneration, while in our studies, the ESTs were obtained from transcriptomes of all tissues at multiple developmental stages and varied physiological conditions found in *A. japonicus*. Second, the two reference libraries included different numbers of original ESTs: ∼7000 ESTs in *H. glaberrima* library and ∼ 30000 ESTs in *A. japonicus* library. Third, the RNA-Seq technique has a higher sensitivity to genes expressed at low or very high levels than DNA microarrays [17]. In a previous study we found 324 up-regulated genes and 80 down-regulated genes during regeneration (body wall at 4dpe and intestine at 7dpe) [22], which is much lower than the number of up-regulated genes (2520) and down-regulated genes (1084) in intestine regeneration at 7dpe. This may be due to the sequencing depth of RNA-Seq (∼5M reads) being larger than that of 454 sequencing (182,473 reads). Moreover, single transcriptome data was insufficient to cover the whole transcriptome, so fewer genes were assembled and recognized. It thus becomes clear that our study is a useful supplement to research carried out on a molecular basis as well as being a more representative collection for studying intestine regeneration.

Gene expression profiles changed with tissue and cellular events during intestine regeneration. During the early stage of intestine regeneration a large number of genes, such as seawi, presenilin, matrix metalloproteinase and Tbx2/3, were abundantly expressed, which contributed to many cellular events, such as cellular migration and differentiation (Table S3, 4). As regeneration proceeded, genes such as helicase, growth arrest-specific 8-like, DnaJ and cyclin-dependent kinase were up-regulated to promote cell proliferation at 7- and14-dpe (Table S4, S5). Lost tissues were then restored and regeneration entered into the intestine growth stage (which included intestinal wall thickening and other processes) so that the number and change fold of DEGs gradually decreased. We have listed the top significant DEGs as a reference for further studies. In our previous transcriptome comparative study [22], genes in the top significant DEGs list, such as proprotein convertase subtilisin/kexin type 9 and cyclin B3, also showed a high degree of differential expression. Orthodenticle, Hu/elav, regeneration-associated protein and speedy A, that were deemed to be associated with regeneration or development in some studies, are good candidate genes for studying intestine regeneration [7], [36], [37]. Nevertheless, many genes in the top DEGs list, such as low-density lipoprotein-related protein, cellular retinol-binding protein, and proprotein convertase subtilisin/kexin type 9, were mainly related to intestinal digestion, absorption, and metabolism. It was thus obvious that their expression levels changed when the intestine lost its function [38], [39], [40]. When this is taken into consideration, it is clear that a search for important regeneration-associated genes should not be strictly confined to the top DEGs as this was not sufficient to search important regeneration-associated genes. To focus on key genes, we compared gene expression between 3dpe and 7dpe stages. Previous studies have demonstrated that the blastema for developing a new intestine forms at 7dpe [8]. The early stage is therefore crucial for regeneration. The method that we used was successful even though only 536 DEGs were observed. Besides H3.3 histone, ribosomal protein L23a and other genes associated with DNA replication, other previously-unnoticed genes attracted our interest (Table 6). For example, it has been demonstrated that annexin could regulate regeneration by affecting the synthesis of EGF [41], [42]. Attractin, a member of the CUB family, plays a crucial role in myelination in the central nervous system, so its over-expression could relate to the reconstruction of the nervous system of regenerative intestine [43], [44]. In addition, over-expression of the solute carrier family and the tetraspanin gene were observed in regenerating rat liver [45], [46]. The genes unique to a certain stage are very important. For example, f-box only protein and heat shock protein 70 were unique to 3dpe (Table S8). F-box protein only mediates the ubiquitination and subsequent proteasomal degradation of target proteins, which play an important role in degrading protein to promote cell migration. Heat shock protein 70 may response stimuli and help sea cucumbers to resist abnormal state. Besides, about two thirds unique genes were “unknown genes” which need further specific research.

### Function classification of DEGs

It is to be expected that the significantly enriched GO terms are mainly associated with development, gene expression, metabolism and cell cycle. In many studies, a number of key developmental regulators have been found to be up-regulated during the regenerative process [2]. Genes mapped to “gene expression” terms, such as Zinc finger protein, ribosomal protein, and Dap5 protein, were activated to produce mature gene products during intestine regeneration. The results also demonstrated some marked trends in gene functioning during the different stages of regeneration. During the early stages, nutrients were rapidly accumulated by genes executing “substance transport”, to provide energy for regeneration and a large number of genes associated with development and cellular events were over-expressed to initiate regeneration. At 7dpe, genes associated with metabolism were activated during the rapid blastema formation stage, when anabolism and catabolism activities (macromolecule biosynthesis and metabolism, protein synthesis and degradation) were dominant [8]. At this stage, intestine wall undergo differentiation, when undifferentiated cells gradually differentiate into different cell types. So anabolism and catabolism activities were dramatic. At the 14dpe stage, there was an obvious change in the expression of genes mapped to “cell cycle”, which was consistent with a previous study in which it was noted that, during this stage, a large number of dividing cells were found in serosa/muscle and mucosa [8]. Besides, at this stage, DGEs were mapped to signal transduction term (signal transduction in response to DNA damage and DNA damage response, signal transduction by p53 class mediator). At the 14dpe stage, the rate of cell proliferation was high, so DNA replication activities was dramatic. Hence, signal transduction was started to response to DNA damage during DNA replication.

### Pathways that play a key role in regeneration

Pathway enrichment analysis identified the most significantly-affected pathways during intestine regeneration. It is no surprise that “Ribosome”, “Spliceosome”, “RNA transport” and“DNA replication” pathways were observed, because protein synthesis is needed for initiating and maintaining regeneration, and the digestion and absorption of vitamin, fat and carbohydrate were inevitably weakened because of the loss of intestine function. The metabolism of Glycerophospholipid, alpha-Linolenic acid and Starch and sucrose were also affected due to hypometabolism (associated with high oxygen consumption) during intestine regeneration [24]. The “Notch signaling pathway” has been reported to play a key role during the regeneration of human intestine epithelia [47], avian retina [48], rat liver [49], zebrafish heart [50] and newt retina [51] by controling multiple cell differentiation, proliferation and apoptotic programs [52]. In the preset study, many genes involved in this pathway – such as delta, notch, histone deacetylase and presenilin – were identified and up-regulated during intestine regeneration. Another noticeable pathway is the “ECM-receptor interaction” pathway. In this pathway, genes – for example, collagen, laminin, tenascin, axonemal dynein and CD36 – that are required for cellular activities such as cell adhesion, migration and differentiation, were also affected [53], [54]. In addition, genes belonging to the FDGF (fibroblast-derived growth factor), TNF (Tumor necrosis factor) and TGF (Transforming growth factor) family were observed to be up-regulated in the “Cytokine-cytokine receptor interaction” pathway. This phenomenon has also been reported during liver regeneration [55], [56].

### Special types of genes associated with regeneration

#### a. Developmental genes

Many developmental genes were differentially expressed during intestine regeneration, Which is not surprising, since regeneration and development are closely related. It appears that developmental genes are reactivated during the regenerative process [4]. Therefore, the developmental genes that we screened were excellent candidates for regeneration study.

The Wnt gene family, which encodes a large group of secreted cysteine-rich proteins, is one of the most intensively studied gene families and is a critical mediator of key cell-cell signaling events during development and regeneration [57], [58]. We found that the expression patterns of *wnt4*, *wnt6* and *wnt8* were not exactly the same during intestine regeneration in *A. japonicus*. As previously reported, distinct Wnt genes have dual roles in regeneration, with some genes promoting regeneration while others inhibited regeneration [59]. The *wnt4* and *wnt6* genes were up-regulated during intestine regeneration of *A. japonicus* and have been shown to be involved in epithelial remodeling, myogenesis, epithelial-mesencheymal transformation and the endomesoderm genes regulatory network [60], [61], [62], [63]. Only a very low level of transcript of *wnt8* was, however, detected during intestine regeneration in *A. japonicus,* indicating that this gene may not contribute to this process. Results from another study have also indicated that *wnt8* transcripts were undetectable by *in situ* hybridization during zebrafish fin regeneration [64].

The Hox genes that encode transcription factors are an important gene family that determines positional identity along the anteroposterior axis in a wide range of metazoans and guides tissue differentiation [65], [66], [67], [68]. These genes determine the progenitor cell fate and positional identity during wound healing and regeneration [66], [67]. As is the case for Wnt genes, the four *hox* genes also show different expression patterns. The *hox1* and *hox3* genes are activated at the early stage of intestine regeneration, while *hox9/10* and *hox 11/13* are stable until a later stage, which is consistent with results from research on *Xenopus* limb regeneration. This research indicated that X*hox*c10 was up-regulated when cells dedifferentiated in the blastema,whereas X*Hox*a13 was slightly re-expressed during later regeneration, while X*Hox*d13 was not expressed until a later regeneration phase [69]. This could be because different Hox genes play distinct roles in regeneration, including wound healing, setting up regeneration, and specifying positional identity [70], [71].

Bone morphogenetic proteins (BMPs) are multi-function growth factors that regulate cell proliferation, survival, differentiation, and apoptosis [72]. An increased level of attention has recently been focused on the role of BMPs in regeneration, such as tail and limb regeneration in *Xenopus* tadpoles [73], newt lens regeneration[74], digit regeneration in fetal mice [75], and bone regeneration in rats [76]. Our results have shown that BMP1 was up-regulated during intestine regeneration, which is consistent with Mashanov et al.' s study on intestine regeneration in *H. glaberrima* [11]. They suggested that Bmp1 may be involved in regulating the folding of the luminal epithelium and gut looping by modulating morphogenetic movements [11].

In our study we found that two other two genes, syndecan and krueppel-like6, were affected by regeneration. These are reported to be associated with muscle and axon regeneration [77], [78]. Syndecans, a member of the transmembrane heparin sulfate proteoglycans (HSPGs) family, are implicated in muscle differentiation and myoblast development to direct muscle regeneration [78], [79], [80]. Krueppel-like factors, a family of zinc finger transcription factors, were deemed to regulate the intricate gene programs associated with axon regeneration [77], [81].

#### b. Extracellular matrix (ECM) associated genes

ECM remodeling related to organ morphogenesis and regeneration, has been demonstrated in many animals: liver regeneration in rats [82], skeletal muscle regeneration in mouse [83], limb regeneration in newts [84], skin regeneration in mammals [85] and intestine regeneration in sea cucumber *H. glaberrima* [13]. Collagen, tenascin, laminin, spondin and fibropellin were similarly observed to be up regulated during intestine regeneration. ECM remodeling is regulated by the proteolytic activities of matrix metalloproteases (MMPs), which degrade ECM components [13], [86]. Many studies have suggested that MMPs are required for tissue remodeling and regeneration, such as newt limb regeneration [87], zebrafish fin regeneration [88], mice epithelial regeneration [89] and skeletal muscle regeneration [90]. Our results also identified up-regulated expression of all MMP genes except MMP14. We have concluded that ECM genes contributed to intestine regeneration in *A. japonicus*.

#### c. Cytoskeletal genes

Myogenesis is one of the most remarkable characteristics of intestine regeneration [35]. Many myocyte events occurred during intestine regeneration of sea cucumbers, including the formation of spindle-like structures, muscle component dedifferentiation, and muscle precursor cell division [35], [91], [92]. Our results showed a dramatic expression variation in cytoskeletal genes. The Cytoskeletal genes- “tubulin” [93], [94], [95], “actin” [96], “myosin” [97] and “gelsolin” [98] have been proved to be related to regeneration. An interesting finding is the down-regulated expression of myosin and gelsolin during intestine regeneration. A similar result was also reported in *H. glaberrima* [7]. The expression of gelsolin was also observed to be down-regulated in regenerating skeletal muscle [99]. This may relate to the observation that there is no muscle layer in the newborn intestine at the early stage of regeneration, so myosin was not needed to regulate muscle motility [8]. Gelsolin has been demonstrated to be a regulator and effector of apoptosis, which would explain why it was down regulated during Cytoskeletal remodeling [100].

## Conclusion

The intestine regeneration of sea cucumbers is a complex process where thousands of genes (more than 15%, ∼3500 ESTs) showed significantly differential expression during intestine regeneration. RNA-Seq can be used for analyzing variantion in gene expression between different samples. Analysis gained by this study will be useful for future regeneration studies on sea cucumbers. Moreover, we demonstrate that intestine regeneration in the sea cucumber is a good model for studies to identify and characterize the molecular basis of organ regeneration.

## Supporting Information

Figure S1 **Sequencing saturation analysis in Normal, 3dpe, 7dpe, 14dpe and 21dpe libraries.**
(TIF)

Table S1 **PCR primers were used for Real-time PCR validation.**
(XLS)

Table S2 **All differential expressed genes at 3dpe Vs 7dpe.**
(XLS)

Table S3 **All differential expressed genes at 3dpe Vs Normal.**
(XLS)

Table S4 **All differential expressed genes at 7dpe Vs Normal.**
(XLS)

Table S5 **All differential expressed genes at 14dpe Vs Normal.**
(XLS)

Table S6 **All differential expressed genes at 21dpe Vs Normal.**
(XLS)

Table S7 **The DEGs (isotigs) in enriched Pathways.**
(XLS)

Table S8 **All genes unique to 3, 7, 14 and 21dpe respectively.**
(XLSX)
