# Correction: RNA-Seq Reveals Dynamic Changes of Gene Expression in Key Stages of Intestine Regeneration in the Sea Cucumber *Apostichopus japonicus*

**DOI:** 10.1371/annotation/d2d71c46-4254-46bd-8a83-9a7a56f2abdf

**Published:** 2013-08-29

**Authors:** Lina Sun, Hongsheng Yang, Muyan Chen, Deyou Ma, Chenggang Lin

There was an error in the title of the article.

The correct title is: RNA-Seq Reveals Dynamic Changes of Gene Expression in Key Stages of Intestine Regeneration in the Sea Cucumber *Apostichopus japonicus*

The correct citation is: Sun L, Yang H, Chen M, Ma D, Lin C (2013) RNA-Seq Reveals Dynamic Changes of Gene Expression in Key Stages of Intestine Regeneration in the Sea Cucumber *Apostichopus japonicus*. PLoS ONE 8(8): e69441. doi:10.1371/journal.pone.0069441 

