# Supplementary figures and images for: RNA-Seq Reveals Dynamic Changes of Gene Expression in Key Stages of Intestine Regeneration in the Sea Cucumber Apostichopus japonicas
Source: PLoS One. 2013 Aug 6;8(8):e69441. doi: 10.1371/journal.pone.0069441 (PMC3735544; doi:10.1371/journal.pone.0069441)

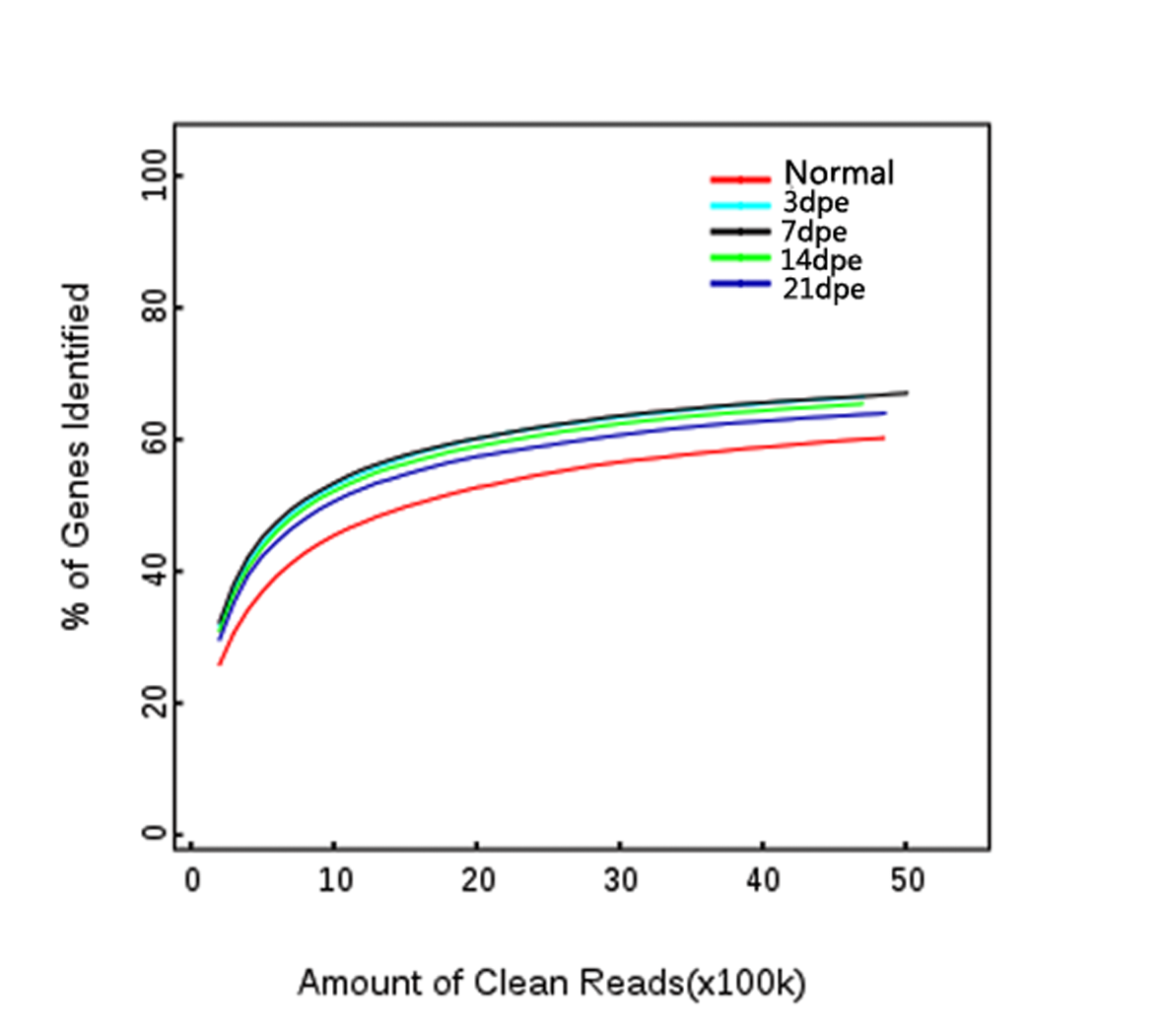

Supplement: Figure S1 — Sequencing saturation analysis in Normal, 3dpe, 7dpe, 14dpe and 21dpe libraries. (TIF) [file pone.0069441.s001.tif]
